# Characterizing Hematological Changes Following Repeated Exposure to Non-Targeted Low-Dose Ionizing Radiation in Prostate Cancer Patients

**DOI:** 10.1177/15593258241276120

**Published:** 2024-10-02

**Authors:** Allison E. Kennedy, Ian S. Dayes, Sameer Parpia, Douglas R. Boreham, Dawn M. E. Bowdish

**Affiliations:** 1 Department of Medicine, Michael G. DeGroot School of Medicine, 12362 McMaster University, Hamilton, ON, Canada; 2 McMaster Immunology Research Center, 12362 McMaster University, Hamilton, ON, Canada; 3 Department of Oncology, 3709 Juravinski Cancer Center, Hamilton, ON, Canada; 4 Department of Oncology, Michael G. DeGroot School of Medicine, Hamilton, ON, Canada; 5 Medical Sciences Division, 26627 Northern Ontario School of Medicine, Sudbury, ON, Canada; 6 Firestone Institute for Respiratory Health, 25479 St Joseph’s Healthcare Hamilton, Hamilton, ON, Canada

**Keywords:** low-dose radiation-treatement, hematology, immunophenotype, leukocyte, monocyte

## Abstract

The duration and magnitude of haematological changes following non-targeted low-dose radiation have not been well explored. We previously reported that low-dose radiation (150 mGy 2x/week for 5 consecutive weeks) was well tolerated by participants (n = 15) with minimal toxicities and no changes in quality of life. Leukocytes, platelets and erythrocytes decreased from baseline measurement 12 months following treatment, however changes were not clinically significant. T-cells, NK-cells, B-cells and neutrophils were found to decrease during treatment and return to baseline levels by 3 months. The monocyte activation marker CD64 (FcγRI) was lower in participants whose cancer did not progress during the 12 month study follow up period, potentially giving insights into a biomarker of treatment success. Herein, we provide one of the most detailed descriptions of hematologic changes during low dose radiation treatment and during one year follow up. Low-dose radiation was associated with minor hematologic changes that mostly resolved by 3 months. (Clinical Trial Registered with the United States National Library of Medicine and National Institutes of Health under the title ‘Low Dose Hemi-Body Radiation for Recurrent Prostate Cancer’; ID: NCT03196778).

## Graphical abstract

**Graphical Abstract:**
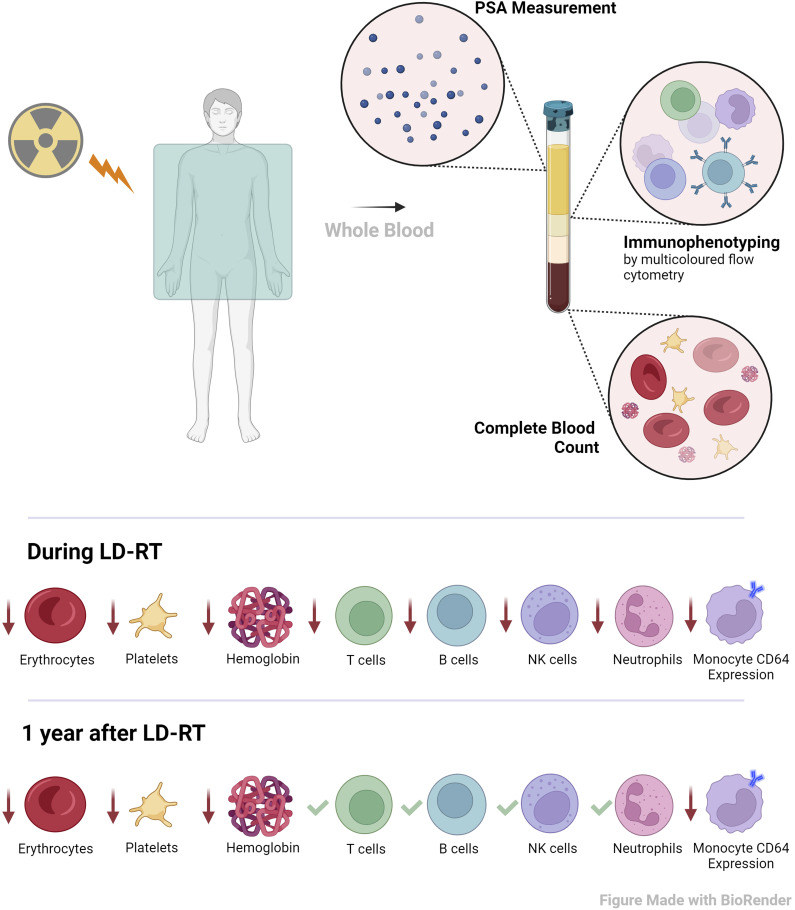

## Introduction

A considerable body of evidence supporting the use of low-dose radiation as a cancer treatment emerged in the 1970s and 1980s.^1–12^ In these early studies, outcomes of total body irradiation (TBI) or hemi-body irradiation (HBI) were monitored in patients with hematological cancers. Although positive outcomes were frequently reported, the same sample size and design of these studies were often flawed. Historical studies report wide variation in dosing patterns for patients within the same study where radiation dose ranged in between 50-500 mGy per fraction delivered anywhere from 3 to 5 times a week. Decrease in leukocyte numbers, platelet numbers and hemoglobin indicative of bone marrow depression, led to halting treatment for patients.^10,11,13–15^ Cumulative doses for each patient within a study would range between 1000-4000 mGy, often falling outside of the range considered to be ‘low-dose’ (<1000 mGy).^2–6^ Herein we deliver a consistent dosing regimen for all participants, to accurately assess immunological alterations from HBI.

Prostate cancer is one of the most frequently diagnosed cancers in men and disease progression is easily monitored by measuring serum prostate specific antigen (PSA).^16^ Primary prostate cancer diagnosis is often treated with localized surgery and/or radiation. If treated effectively, PSA levels are low or undetectable after treatment. Patients are routinely monitored for rising PSA levels as more than a third of patients will have their cancer recur within 10 years.^17^ When PSA levels rise, the treatment options may impact quality of life, introducing potential for non-toxic low-dose radiation therapy to eliminate those negative options. In a single case study, Kojima et al^18^ reported that a patient with recurrent prostate cancer whose PSA declined from >5 mg/mL to near non-detectable levels (0.085 mg/mL) after 6 weekly fractions of 150 mGy of radiation. PSA was reported to remain reduced for another 24 weekly fractions, but the authors did not report whether PSA changes after fractions ceased.

The number and efficacy of cancer therapies over the last decade has significantly increased and yet managing a cancer diagnosis still poses high number of symptoms and stressors on patients. When considering options, patients are faced with decision factors including treatment efficacy, overall survival and changes in quality of life during treatment, long after treatment has finished. In addition to this, cancer therapies require visiting major medical centers for repeated visits over several months, posing a financial burden. As a possible emerging cancer therapy, quality of life in addition to acute and long-term hematological changes need to be considered when exploring low-dose radiation treatment (LD-RT). To date, use of low-dose radiation has identified some potential hematological toxicities associated with treatment, such as decreases in platelet and leukocyte numbers^2,3,11^ however the risk factors associated with these effects nor the longevity of these outcomes have not been well documented. Our study was designed to fully characterize the short-term and long-term hematological changes, during a 5-week low dose radiation regime for treating recurrent prostate cancer.

## Methods and Materials

### Participant Recruitment

Patients with recurrent prostate cancer were recruited from a single institution between September 2017 and November 2019. The study protocol and all supporting documents were approved by the local hospital and Hamilton Integrated Research Ethics Board (HiREB #2706) and was registered with the United States National Library of Medicine and National Institutes of Health (#NCT03196778). To be eligible, participants must have had a histologic diagnosis of adenocarcinoma of the prostate, have had either prior prostate surgery, radiation therapy or both, shown evidence of recurrence in the disease by rising circulating PSA levels (nadir +2 ng/mL), and had PSA values recorded for at least a year prior to enrollment. Patients who had received prior treatment with chemotherapy, abiraterone, enzalutamide, or radium-223; were taking any immunosuppressive medications; or had a platelet count <50 × 10^9^/L, a leukocyte count <3 × 10^9^/L, or a granulocyte count <2 × 10^9^/L were excluded. Once deemed eligible, informed consent was obtained.

### Sample Size

Sample size was calculated based on a Simon 2-stage design study. Sample size estimation was calculated assuming the probability of response under the null hypothesis of *P* < .1, and *P* < .25 under the alternate hypothesis (alpha, 0.15; power, 0.80), and the maximum sample size required was determined to be 21 patients. After 16 participants completed the study (stage 1), if ≤ 1 participants responded, the trial would be terminated. If > 1 participant responded in stage 1, then the trial would continue to enroll an additional 5 patients. With the completion of 21 patients, therapy is to be rejected if the total number of responding patients is ≤ 3.

### Radiation Intervention

Each participant received 10 doses of radiation at Juravinski Cancer Center in Hamilton, ON. Treatments were scheduled twice a week for 5 weeks with at least two days separating each session. Each dose of 150 mGy was delivered to a half-body field, spanning from the participants thyroid to mid-thigh, at a dose rate of 1 Gy/min using 6 MV Xrays as previously published.^19^ Each participant received a cumulative weekly dose of 300 mGy and a total study dose of 1500 mGy. Treatment dose was verified in the first two participants enrolled in the study with thermoluminescent dosimetry.

### PSA Measurement

The primary outcome of this study was to determine the efficacy of LD-RT as a treatment for recurrent prostate cancer by decreasing PSA by 50%. Blood was collected at the pre-treatment study visit and the first day of LD-RT before receiving radiation to determine eligibility and capture baseline PSA. Blood was similarly collected at the last day of LD-RT and at 1, 3, 6 and 12 months after treatment to determine longevity of PSA responses. PSA responses were previously published.^19^

### Hematological Measurements by Complete Blood Count

Venous blood in heparin tubing (Thermo Fisher Scientific, Waltham, MA, USA) was collected at the pre-treatment study visit and the first day of LD-RT, before receiving radiation, to determine eligibility and capture baseline hematological parameters. Complete blood counts were performed at the core lab at Juravinski Cancer Center in Hamilton ON. Blood was also collected at the beginning of each treatment week, before radiation treatment, to monitor hematological parameters over the duration of the study, and at 1, 3, 6 and 12 months after treatment to monitor long-term status of hematological parameters follow LD-RT. As previous studies reported haematological toxicities,^2,3,11^ treatment was to be withheld if platelet count was <50 × 10^9^/L, leukocyte count was <3 × 10^9^/L or granulocyte count was <2 × 10^9^/L at the beginning of each treatment week.

### Peripheral Immunophenotyping

Venous blood in heparin tubing (Thermo Fisher Scientific, Waltham, MA, USA) was collected at the same time as blood needed for complete blood counts to quantitate circulating neutrophils, monocytes, T, B and NK cells by multicolour flow cytometry as previously described^20,21^ at McMaster University in Hamilton ON. Direct application of monoclonal antibodies (specificities outlined in Supplemental Table 1) to 100 μL of whole blood for 30 minutes at room temperature. Following staining, samples were incubated with either 1 × Fix/Lyse Buffer (eBioscience, Thermo Fisher Scientific, Waltham, MA, USA) for 10 minutes or following standard protocols for the FOXP3 Transcription Factor Staining Kit (eBioscience, Thermo Fisher Scientific, Waltham, MA, USA), washed with PBS, and resuspended in FACS Wash (5 mM EDTA, 0.5% BSA in PBS) for analysis with a LSRII (4 laser, BD Biosciences, Franklin Lakes, NJ, USA). Absolute counts for circulating hematological populations were determined using CountBright™ absolute counting beads (Invitrogen Life Technologies, Carlsbad, CA, USA). Gating strategies and representative FACS dot plots to determine circulating hematological populations are shown in Supplemental Figure 1.

### Statistical Analysis

Data and statistical analyses were done in FlowJo (Version 10.8.1, Ashland, OR, USA), GraphPad Prism (Version 9.0.1, San Diego, CA, USA), and R (open-source software). Multiple group comparisons were tested using Welch’s One-Way ANOVA and Tukey’s multiple comparisons post-hoc test.

## Results

### Participant Demographics

Participants were 76.5 ± 7.3 years of age and were primarily white (93.8%). A full overview of participant demographics can be reviewed in Dayes et al, 2023.^19^ The mean time since the initial cancer treatment to enrolling in our study was 8.4 ± 3.5 years. Most of our participants received radiation therapy as the primary treatment for their initial prostate cancer diagnosis (n = 10; 62.5%). Approximately a third (n = 5; 31.3%) of the participants previously had a radical prostatectomy in conjunction with radiation therapy, and 1 participant received brachytherapy. At the time of consent, 5 participants (31.3%) were currently undergoing androgen deprivation therapy for their prostate cancer.

### Study Compliance

Study compliance was high, with 15 of 16 (93.8%) patients completing the 5-week treatment. One participant with an extensive cardiac history experienced a significant cardiac event during LD-RT resulting in death. This event was reported to the local ethics committees in addition to being reviewed by an independent Data Safety Monitoring Board and was determined to have not been related to LD-RT treatment.

### Hematological Changes With LD-RT

**Figure 1.**
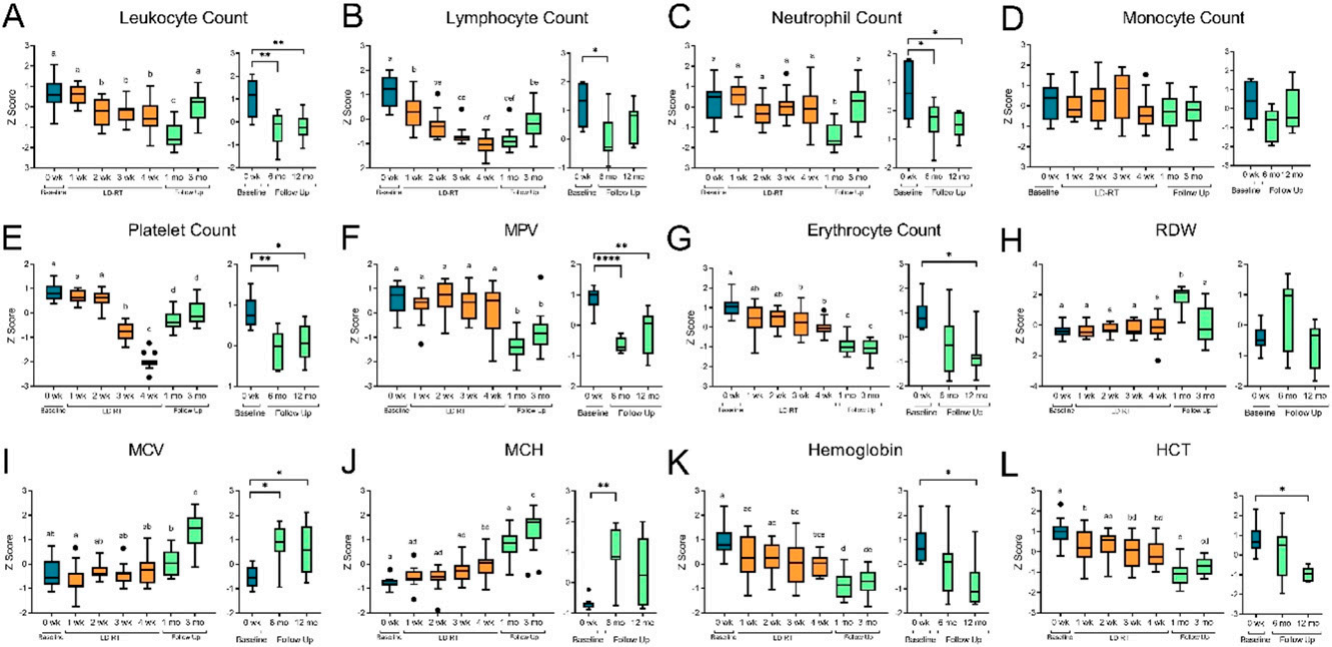
Hematological parameters changed during LD-RT and persisted for 12 months. Pre-treatment data shown in blue (n = 15), during LD-RT (5 weeks) shown in orange (n = 15) and follow up data shown in green (1, 3 months n = 15). Participants starting salvage therapy were removed from follow up analysis at 6 and 12 months (n = 7) Data shown in Tukey method box and whisker plots. Box limits show the 25^th^ to 75^th^ percentiles with the line within in box representing the median. Multiple group comparisons were tested using Welch’s One-Way ANOVA and Tukey multiple comparison correction post-hoc tests. *P < .0332, **P < .0021, ***P < .0002, ****P < .0001.

As expected, leukocyte counts significantly decreased in peripheral blood after only 2 weeks of LD-RT (see Supplemental Table 2 for raw numbers). Leukocytes numbers continue to decrease for 1 month after treatment, but return to pre-treatment numbers 3 months after LD-RT (Figure 1A). Leukocyte counts were monitored weekly, and remained above the safety threshold of 3 × 10^9^/L in all participants. The decrease in leukocyte count during radiation treatment was attributed to the decreasing numbers in circulating lymphocytes (Figure 1B), as neutrophil and monocyte numbers were conserved over the 5-week treatment (Figure 1C and D). The number of neutrophils in circulation were decreased from pre-treatment values and remained lower up to 12 months following radiation. At no point was the decrease in neutrophils considered to be a clinically significant neutropenia (<2 × 10^9^/L) (Supplemental Figure 2).

Platelets are the most radiosensitive hematological parameter^22^ and were monitored weekly. Platelet counts remained above the study safety threshold of 50 × 10^9^/L, however thrombocytopenia was observed in 80% (n = 12) of participants after 3 or 4 weeks of treatment (Supplemental Figure 2). Twelve months following the last radiation dose, platelet numbers were still statically lowered than baseline measurements, but were higher than numbers recorded during LD-RT. Mean platelet volume (MPV), a measurement to determine the average size of platelets found in the blood, showed no alterations in platelet size during the 5 weeks of radiation. A significant decrease in MPV from clinical normal range was observed in many participants 1 month after radiation. This change in platelet size did not resolve by 12 months after radiation (Supplemental Figure 2).

Erythrocyte count significantly decreased after 3 weeks of radiation and did not recover by the 12 month follow up visit (Figure 1G). Hemoglobin and hematocrit (HCT) levels, which is a measurement of the percentage of blood that is red blood cells decreased, consistent with the loss of erythrocytes in circulation (Figure 1K and L). To our surprise, other red blood cell measurements such as mean corpuscular volume (MCV), a measurement of the average size of the red blood cells, mean corpuscular hemoglobin (MCH) a measurement of the average amount of hemoglobin in a red blood cell, and red cell distribution width (RDW) measure larger erythrocytes following treatment (Figure 1H and J). Prior to starting radiation, 4 participants had lower than normal erythrocyte numbers which persisted during treatment, however MCV, MCH and RDW remained within normal ranges (Supplemental Figure 2).

**Figure 2.**
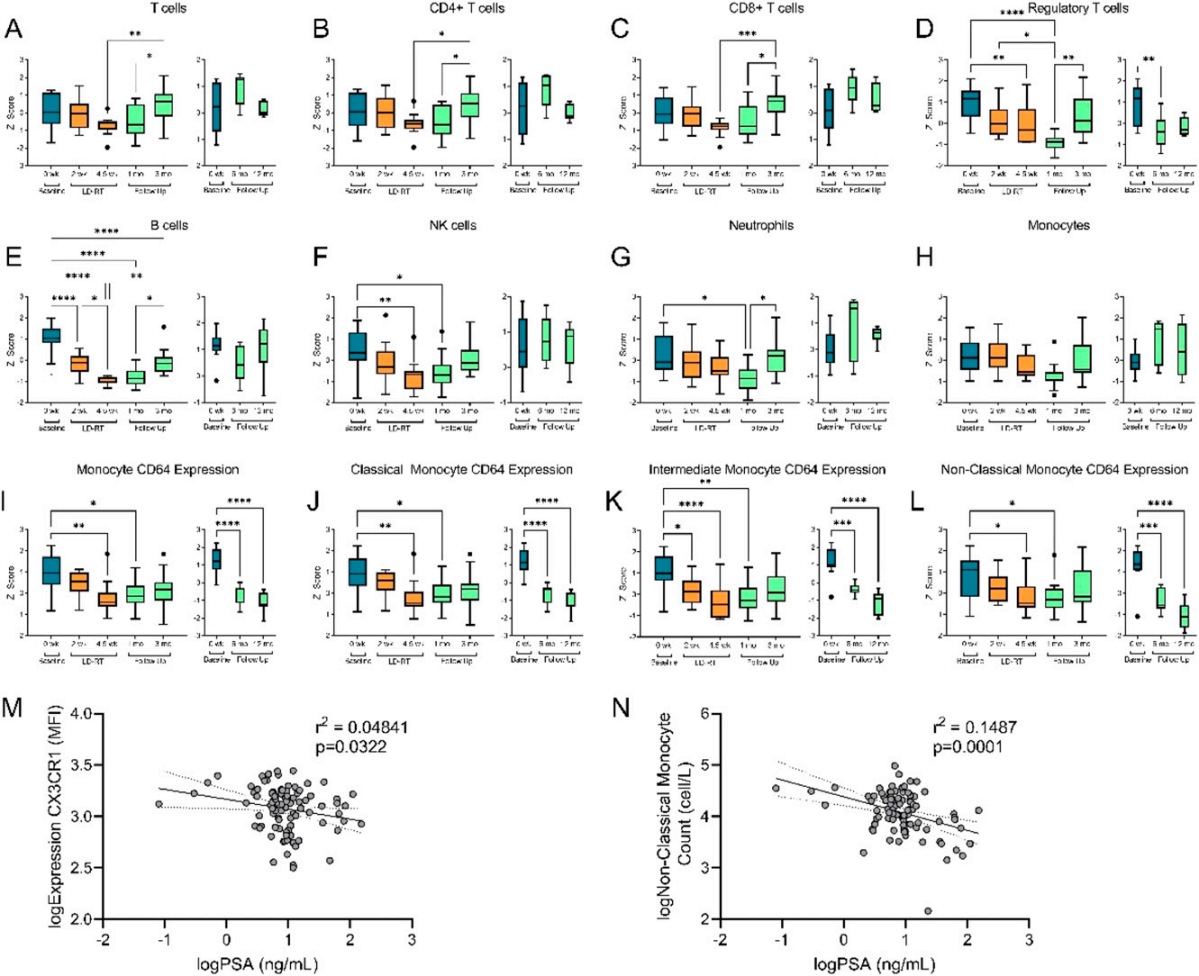
Changes in peripheral immunophenotype during LD-RT and persisted 3-12 months. (A-L) Pre-treatment data shown in blue (n = 15), during LD-RT shown in orange (n = 15) and follow up data shown in green (1, 3 months n = 15). Participants starting salvage therapy were removed from follow up analysis at 6 and 12 months (n = 7) Data shown in Tukey method box and whisker plots. Box limits show the 25^th^ to 75^th^ percentiles with the line within in box representing the median. Multiple group comparisons were tested using Welch’s One-Way ANOVA and Tukey multiple comparison correction post-hoc tests. *P < .0332, **P < .0021, ***P < .0002, ****P < .0001. Spearman’s correlation of absolute PSA and classical monocyte expression of CX_3_CR_1_ (M), and absolute non-classical monocytes (N).

To help give further insights into changes in leukocyte populations, peripheral blood immunophenotyping was performed. Absolute T cell counts, CD4^+^ T cells and CD8^+^ T cells did not show any significant decreases in circulation during the 5-week radiation treatment (Figure 2A-C). B cells which are known to be more radiosensitive, decreased starting as early as 2 weeks into treatment, requiring 6 months after the last dose of radiation to return to pre-treatment levels (Figure 2E). Another radiosensitive population, NK cells, significantly decreased during treatment (Figure 2F) but recovered to pre-treatment levels by 3 months. Regulatory T cells, which are positively associated with prostate cancer,^23^ were shown to decrease during radiation (Figure 1D). Some participants required salvage hormone therapy 3 months after LD-RT as their PSA continued to rise. In patients with a stable disease (did not need to start salvage therapy), regulatory T cells were shown to be lower than baseline measurements until 12 months after treatment, suggesting a possible indicator of disease stability. Consistent with CBC measurements, no changes were observed in neutrophil or monocyte numbers during radiation (Figure 2G and H).

**Figure 3.**
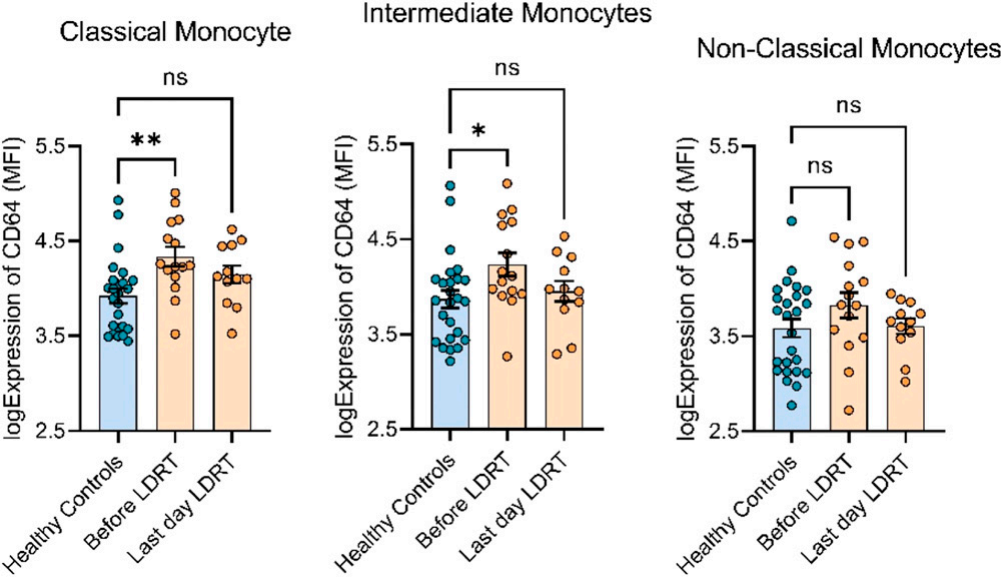
CD64 expression on circulating classical, intermediate and non classical monocytes. A healthy control population (n = 25, mean (SD) age = 70.1 ± 14.9) shown in blue, compared to CD64 expression in prostate cancer patients (n = 15, mean (SD) age = 76.5 ± 7.3) at baseline, and at the end of LD-RT. At baseline, prostate cancer patients have increased expression of CD64 compared to healthy controls on classical and intermediate monocytes. This increase in expression no longer exists after LD-RT. Multiple group comparisons were tested using Welch’s One-Way ANOVA and Tukey multiple comparison correction post-hoc tests. *P < .05; **P < .01; ****P < .0001.

Monocytes are a sensitive marker of inflammation contributing to pro- and antitumoral immunity.^24^ We assessed monocyte subsets for expression of migratory and activation makers during LD-RT. Expression of CD64 (FcγRI), a high affinity receptor for IgG and a receptor associated with monocyte activation, significantly decreased by the end of therapy (Figure 2I-L) and in participants with stable disease, remained decreased until at least 12 months following treatment. Baseline CD64 expression on classical and intermediate monocytes were elevated in prostate cancer patients compared to a healthy age, sex matched controls (Figure 3). This elevation in CD64 expression was eliminated by the end of LD-RT, identifying a potential biomarker for progressing prostate cancer.

Classical monocytes expressing CCR2 are first to leave the bone marrow and are either recruited to sites of acute inflammation, or differentiate into CX_3_CR_1_ expressing intermediate monocytes.^25^ We saw no differences in expression of CCR2 or CX_3_CR_1_ as a result of LD-RT, however we measured a decrease in CX_3_CR_1_ expression on classical monocytes as absolute PSA decreased (Figure 2M). CX_3_CR_1_ is associated with recruitment to tissues or vasculature and repair of damage in response to CX_3_CR_1_/fractalkine.^26^ A decrease in CX_3_CR_1_ on classical monocytes suggests that the monocytes with the highest levels of CX_3_CR_1_ have emigrated from the circulation in response to the progression of prostate cancer (increased PSA). This is further supported with the negative relationship measured between non-classical/patrolling monocytes and absolute PSA (Figure 2N).

## Discussion

This study aimed to characterize the hematologic effects of LD-RT in effort to better understand transient and long-term hematological changes. LD-RT may have potential as a cancer therapy, however it is not clear whether potential hematological toxicities, especially in potentially vulnerable cancer patients occurs.

As previously reported, our primary criterion for this study was to decrease PSA levels by 50% as the clinical marker for disease progression.^19^ Without intervention, when PSA levels reach a level that suggest prostate cancer recurrence (nadir +2 ng/mL), one would expect the patient will continue with that trajectory of progression. Although the primary criterion for this study was not met, 3/16 participants had their PSA levels plateau for at least 12 months after LD-RT with no change in their quality of life.^19^ An observed plateau of PSA progression would suggest a potential cytostatic response to LD-RT and likely led to delaying the need for further treatments. Rather than a curative therapy, our findings suggest LD-RT as a promising and readily available method for managing PSA kinetics in patients to delay the need to initiate the next line to treatment. For most men in our study, the next line of treatment would have been androgen deprivation therapy, which is associated with a long list of unwanted side effects and a decrease in quality of life.

While high dose radiation therapy is generally performed with targeted beams, reports of LD-RT is typically non-targeted and include a large amount of the body, and large sections of bone marrow. Hematological abnormalities such as thrombocytopenia, leukopenia and a decrease in granulocytes have been commonly reported in studies using LD-RT, and severe enough to halt scheduled therapy. Interpreting these abnormalities is complicated when patients in these studies are currently taking or have recently finished chemotherapy agents, which is known to suppress the immune system. Our data show that leukocyte numbers significantly decrease after 600 mGy of radiation but do not lead to long term consequences, as 3 months after radiation they return to pre-treatment levels. Platelet count was also significantly affected during LD-RT and did not return to pre-treatment levels by 12 months. Although not as pronounced, erythrocyte number and size were also affected 12 months following radiation. It is important to note that this decrease was relatively mild with no observed clinical consequence.^19^

Hematological toxicities associated with high-dose radiation therapy differ from those observed in LD-RT, as LD-RT primarily reports most severely impacting thrombocytes whereas reports using high-dose radiation largely report severe toxicities regarding leukocyte population. A review of hematological toxicities after whole pelvis radiation revealed that leukopenia (clinical grade 2 (<2.9 × 10^9^/L)) occurred in 10.2% of patients^27^; grade 2 granulocyte (<1.4 × 10^9^/L) and hemoglobin (<9.4 g/dL) toxicity also occurred in 1.9% and 1.2% of patients respectively.^27^ Miszczyk and Majewski^28^ describe the treatment of 115 patients with prostate or bladder cancer through definitive radical radiation therapy. All 74 prostate cancer patients received 76 Gy to the primary tumour while the patients with bladder cancer received a range of 60-70 Gy. Leukocyte counts were reduced by 33% over the course of treatment, with neutrophils decreased by 24%. Lymphocyte counts had the greatest reduction with a mean decrease of 62% and was severe enough to result a grade 3 toxicity (as measured by Common Terminology Criteria for Adverse Effects (CTCAE) v4.0) in 19% of patients. In another study,^29^ 10 patients received 30-40 Gy to the pelvis and paraaortic lymph nodes for either testicular or ovarian malignancies. Lymphocytes were impacted the most, declining from 2.4 × 10^3^/μL at the beginning of treatment to 0.6 × 10^3^/μL. After 12 months lymphocyte counts were still significantly lower than pre-treatment at less than 60% of starting values and a mean of 1.4 × 10^3^/μL. Platelet counts also dropped by approximately 60% from an initial mean of 315 × 10^3^/μL to 195 × 10^3^/μL. After 12 months platelet counts remained significantly lower than pre-treatment values at 227 × 10^3^/μL. In general, the severity of hematological effects from high-dose radiation therapy is dependent on the location and the tissues involved in the treatment field, with more severe toxicities reported when including a large area of bone marrow. Overall, the hematological toxicities from high-dose radiation are more severe and show evidence of leaving the patients immune compromised for longer compared to the hematological effects observed in our study.

It has been proposed that LD-RT may increase the number of leukocytes in the circulation and thus improving treatment outcome.^30^ One study of Non-Hodgkin’s Lymphoma demonstrated a transient increase in CD4^+^ T cells and an increase in CD4+/CD8 + ratio.^13^ We did not observe increase numbers of any of the leukocyte populations measured. These findings highlight a key window of time necessary for desired outcomes as we measure immune populations 4-5 days after a patient’s last dose of radiation. Increasing the frequency of low dose treatments, lowering the dose, and consequently shortening the time between exposures may enhance and maintain the magnitude of the cellular responses. A second, smaller sub-study is currently underway to explore this possibility.

Monocytes are responsible for antigen recognition and repair of tissues and vessels after damage. Functional changes in circulating monocytes may provide useful information about the biological features of disease progression, and yet are not often considered as a population to measure in clinical studies. One study reported no alteration in monocyte numbers due to LD-RT^13^ however to our knowledge, there are no studies investigating monocyte function after exposure to LD-RT. Increased levels of CD64 (Fcγ-receptor 1) have been associated with monocyte activation and found to be elevated in cancer patients relative to normal donors.^31,32^ Our data demonstrate that treatment with low-dose radiation reduced CD64 expression on monocytes in circulation. Whether CD64 expression is a biomarker of disease progression or responsiveness to treatment remains to be seen.

Limitations of this study include the lack of a control group and differences in patient androgen deprivation therapy status. These two points contribute to some heterogeneity within the study population hampering the strength of our findings. In addition, this study was performed at a single institution limiting generalizability. With a small number of subjects and relatively short follow-up, our primary outcome was limited to PSA response and does not address more important clinical endpoints, such as overall survival or time to metastatic disease. The results from this study would be strengthen with validation studies performed by another independent group. All subjects were asymptomatic at time of enrollment, so there was no opportunity to observe improvements in symptoms, performance status, or quality of life, although there was no evidence of a reduction in the latter. Due to the lack of diversity within the sample population, we cannot confidently assess if age, lifestyle factors, the status of disease, or previous history with radiation treatments impacts the hematological changes observed. A larger study would be required fully assess the impacts of these factors. This study also does not well address the role of such a treatment in the ever-expanding armamentarium against recurrent prostate cancer. However, from our results, any value it may have likely lies in the early phase of recurrence, before the use of androgen deprivation therapy, to delay both the commencement of androgen deprivation and the initiation of castrate resistance. Whether the systemic nature of this treatment limits any future role for cytotoxic therapy remains unknown.

## Conclusion

Our study is the first to carefully examine the hematological changes that occur during a 5-week treatment of 150 mGy of LD-RT twice per week in prostate cancer patients, in addition to monitoring hematologic parameters for 12 months. We previously reported this treatment has minimal toxicities, no changes in quality of life and was able to delay the initiation of conventional salvage therapy for at least 12 months in 8 out of 15 participants.^19^ Our data show that hematologic changes were relatively short-lived with the exception of monocyte expression of CD64, which can potentially be viewed as a marker of prostate cancer progression. With further research and optimization, LD-RT has the potential to become an effective treatment option for managing recurrent prostate cancer and possibly other forms of malignant disease.

## Supplemental Material

Supplemental Material - Characterizing Hematological Changes Following Repeated Exposure to Non-Targeted Low-Dose Ionizing Radiation in Prostate Cancer Patients Supplemental Material for Characterizing Hematological Changes Following Repeated Exposure to Non-Targeted Low-Dose Ionizing Radiation in Prostate Cancer Patients by Allison E. Kennedy, Ian S. Dayes, Sameer Parpia, Douglas R. Boreham, and Dawn M. E. Bowdish in Dose-Response
