# Supplementary material for: Characterizing Hematological Changes Following Repeated Exposure to Non-Targeted Low-Dose Ionizing Radiation in Prostate Cancer Patients
Source: Dose Response. 2024 Oct 2;22(4):15593258241276120. doi: 10.1177/15593258241276120 (PMC11503843; doi:10.1177/15593258241276120)
Supplement: Supplemental Material - Characterizing Hematological Changes Following Repeated Exposure to Non-Targeted Low-Dose Ionizing Radiation in Prostate Cancer Patients [file sj-pdf-1-dos-10.1177_15593258241276120.pdf]

Supplemental Information

Supplemental Table 1: Fluorophore-conjugated antibodies used for flow cytometry

Cell Surface Marker	Fluorophore	Clone	Company	Cat No.	Antibody Dilution
**Lymphoid staining**					
**CD3**	APCeF780	UCHT1	eBioscience[1]	47-0038-41	1/200
**CD4**	PerCPCy5.5	OKT4	eBioscience[1]	45-0048-42	1/100
**CD8**	PE-Cy7	RPA-T8	BD Pharmingen[2]	557750	1/50
**CD45**	BV510	HI30	BioLegend[3]	304036	1/100
**CD56**	PE	5.1H11	BioLegend[3]	362524	1/50
**NKp46**	PE	9-E2	BD Biosciences[4]	557991	1/50
**CD19**	AF700	HIB19	eBioscience[1]	56-0199-42	1/50
**Myeloid staining**					
**CD45**	BV510	HI30	BioLegend[3]	304036	1/100
**CD16**	PE-Cy7	CB16	eBioscience[1]	25-0168-42	1/100
**CD14**	BV421	M5E2	BioLegend[3]	301830	1/100
**CCR2**	PE	K036C2	BioLegend[3]	357205	1/50
**CD11b**	APC	ICRF44	BD[5]	561051	1/50
**HLA-DR**	PerCPCy5.5	LN3	eBioscience[1]	45-9956-42	1/100
**CX3CR1**	FITC	2A9-1	Cedarlane[6]	D070-4	1/50
**CD15**	BV650	SSEA-1	BioLegend[3]	323033	1/200
**CD3**	AF700	UCHT1	BD[5]	557943	1/50
**CD56**	AF700	5.1H11	BioLegend[3]	362522	1/50
**CD19**	AF700	HIB19	eBioscience[1]	56-0199-42	1/50
**Myeloid Maturity staining**					
**CD45**	BV510	HI30	BioLegend[3]	304036	1/100
**CD16**	PE-Cy7	CB16	eBioscience[1]	25-0168-42	1/100
**CD14**	BV421	M5E2	BioLegend[3]	301830	1/100
**CD13**	PE	WM15	BioLegend[3]	301703	1/100
**CD115**	APC	9-4D2-1E4	BioLegend[3]	347306	1/50
**HLA-DR**	PerCPCy5.5	LN3	eBioscience[1]	45-9956-42	1/100
**CD64**	BV605	10.1	BioLegend[3]	305034	1/50
**CD3**	AF700	UCHT1	BD[5]	557943	1/50
**CD56**	AF700	5.1H11	BioLegend[3]	362522	1/50
**CD19**	AF700	HIB19	eBioscience[1]	56-0199-42	1/50
**Regulatory T cell staining**					
**CD45**	BV510	HI30	BioLegend[3]	304036	1/100
**CD3**	AF700	UCHT1	BD[5]	557943	1/33

**CD4**	PerCPCy5.5	OKT4	eBioscience[1]	45-0048-42	1/100
**CD8**	PECy7	RPA-T8	BD Pharmingen[2]	557750	1/100
**CD25**	PE	BC96	eBioscience[1]	12-0259-41	1/17
**CD127**	APC	eBioRDR5	eBioscience[1]	17-1278-41	1/20
**FoxP3**	FITC	236A/E7	eBioscience[1]	11-4777-41	1/10

[1] eBioscience, Thermo Fisher Scientific, Waltham, MA, USA
[2]BD Pharmingen, Franklin Lakes, NJ, USA
[3]BioLegend, San Diego, CA, USA
[4]BD Biosciences, Franklin Lakes, NJ, USA
[5]BD, Franklin Lakes, NJ, USA
[6]Cedarlane, Burlington, ON, Canada

Supplemental Figure 1: Flow Cytometry Gating Strategies

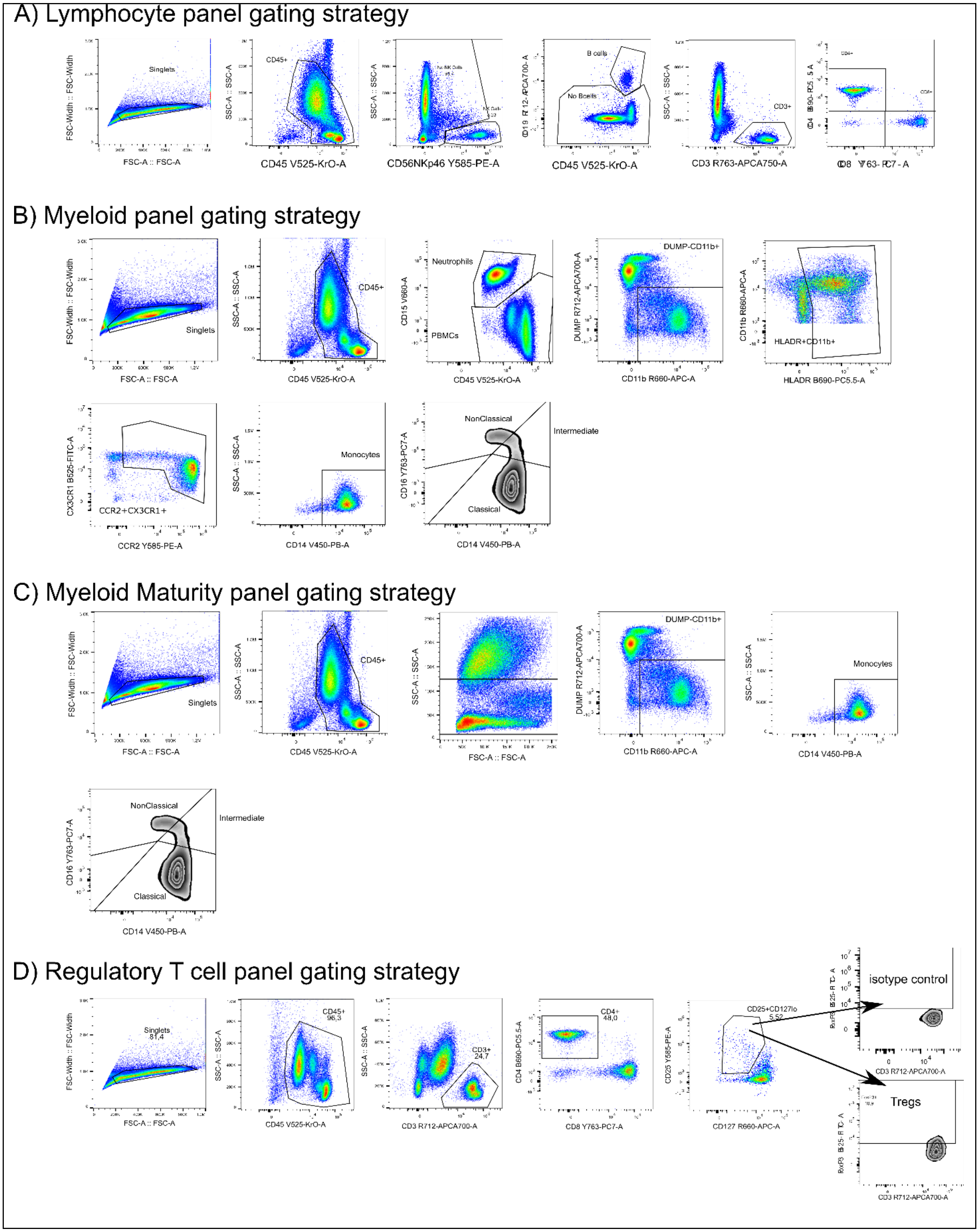

Supplemental Table 2: Comparison of complete blood count parameters and leukocyte populations during LD-RT and for 12 months afterwards.

	During LD-RT n=15								Follow-up Analysis† n=7			
**Complete Blood Count**	**Baseline mean (SEM)**	**1 wk LD-RT mean (SEM)**	**2 wk LD-RT mean (SEM)**	**3 wk LDRT mean (SEM)**	**4 wk LD-RT‡ mean (SEM)**	**1 mo Follow Up mean (SEM)**	**3 mo Follow Up mean (SEM)**	**R squared**	**Baseline mean (SEM)**	**6 mo Follow up mean (SEM)**	**12 mo Follow up mean (SEM)**	**R squared**
Leukocyte Count (x10^9/L)	6.83 (0.31)	6.83 (0.4)	5.92 (0.33)	5.98 (0.37)	5.79 (0.36)	4.8 (0.39)	6.26 (0.34)	0.50****	7.16 (0.48)	5.87 (0.35)	6.06 (0.38)	0.48**
Lymphocyte Count (x10^9/L)	1.66 (0.14)	1.47 (0.19)	1.24 (0.12)	1.07 (0.09)	0.98 (0.09)	1.07 (0.15)	1.26 (0.11)	0.65****	1.47 (0.11)	1.19 (0.14)	1.4 (0.21)	0.35*
Neutrophil Count (x10^9/L)	4.19 (0.27)	4.37 (0.27)	3.69 (0.22)	3.97 (0.27)	3.92 (0.27)	2.95 (0.26)	4.09 (0.29)	0.35****	4.61 (0.43)	3.79 (0.27)	3.71 (0.22)	0.39*
Monocyte Count (x10^9/L)	0.69 (0.05)	0.68 (0.05)	0.72 (0.06)	0.71 (0.05)	0.65 (0.05)	0.62 (0.05)	0.65 (0.04)	0.09	0.77 (0.07)	0.66 (0.05)	0.71 (0.04)	0.19
Platelet Count (x10^9/L)	225 (20.61)	217.2 (18.3)	212.6 (15.37)	165.27 (13.89)	122.13 (10.17)	176.54 (14.53)	188 (12.27)	0.88****	194.43 (15.42)	167.86 (13.89)	170 (14.11)	0.47**
MPV (fL)	10.01 (0.19)	9.93 (0.2)	10.06 (0.22)	9.93 (0.17)	9.88 (0.19)	9.25 (0.19)	9.49 (0.18)	0.52****	10.13 (0.26)	9.57 (0.24)	9.71 (0.28)	0.65****
Erythrocyte Count (x10^12/L)	4.83 (0.12)	4.71 (0.11)	4.7 (0.13)	4.63 (0.14)	4.59 (0.13)	4.36 (0.15)	4.34 (0.11)	0.63****	4.75 (0.16)	4.49 (0.12)	4.39 (0.16)	0.39*
RDW (%)	13.09 (0.15)	13.12 (0.17)	13.16 (0.16)	13.17 (0.14)	13.21 (0.15)	14.08 (0.16)	13.37 (0.2)	0.53****	13.06 (0.19)	13.21 (0.24)	13 (0.18)	0.19
MCV (fL)	93.51 (0.52)	93.24 (0.53)	93.9 (0.4)	93.71 (0.46)	93.99 (0.51)	94.33 (0.48)	96.91 (0.52)	0.58****	93.21 (0.81)	95.86 (1.21)	95.71 (1.27)	0.38*
Hemoglobin (g/L)	146.73 (3.44)	143.67 (3.03)	143.47 (3.81)	141.8 (4.04)	141.4 (3.93)	136.23 (4.4)	137.73 (3.83)	0.42****	145.14 (3.93)	140.71 (2.69)	136.43 (3.26)	0.32*
HCT	0.45 (0.01)	0.44 (0.01)	0.44 (0.01)	0.43 (0.01)	0.43 (0.01)	0.41 (0.01)	0.42 (0.01)	0.49****	0.44 (0.01)	0.43 (0.01)	0.42 (0.01)	0.39*
**Absolute Count**	**Baseline mean (SEM)**	**1 wk LD-RT mean (SEM)**	**2 wk LD-RT mean (SEM)**	**3 wk LDRT mean (SEM)**	**4.5 wk LD-RT‡ mean (SEM)**	**1 mo Follow Up mean (SEM)**	**3 mo Follow Up mean (SEM)**	**R squared**	**Baseline mean (SEM)**	**6 mo Follow up mean (SEM)**	**12 mo Follow up mean (SEM)**	**R squared**
T cells	1.52 (0.28)	n/ap	1.57 (0.32)	n/ap	1.17 (0.2)	1.12 (0.26)	1.82 (0.23)	0.21**	1.17 (0.27)	1.46 (0.27)	1.37 (0.2)	0.24*
CD4+ T cells	0.99 (0.15)	n/ap	0.96 (0.16)	n/ap	0.77 (0.11)	0.74 (0.16)	1.16 (0.16)	0.19**	0.84 (0.17)	1.06 (0.23)	0.92 (0.11)	0.23
CD8+ T cells	0.43 (0.12)	n/ap	0.5 (0.15)	n/ap	0.32 (0.09)	0.32 (0.09)	0.55 (0.11)	0.23**	0.24 (0.1)	0.31 (0.09)	0.34 (0.09)	0.25*
Regulatory T cells	0.04 (0.01)	n/ap	0.03 (0.01)	n/ap	0.03 (0.01)	0.01 (0)	0.04 (0.01)	0.37****	0.04 (0.01)	0.03 (0.01)	0.02 (0)	0.41**
B cells	0.17 (0.04)	n/ap	0.09 (0.02)	n/ap	0.02 (0.01)	0.03 (0.01)	0.08 (0.02)	0.66****	0.11 (0.05)	0.07 (0.02)	0.19 (0.09)	0.17
NK cells	0.61 (0.12)	n/ap	0.51 (0.09)	n/ap	0.37 (0.1)	0.47 (0.21)	0.61 (0.14)	0.24***	0.48 (0.09)	0.67 (0.1)	0.78 (0.14)	0.01
Neutrophils	5.86 (0.81)	n/ap	6 (1.24)	n/ap	4.82 (0.83)	3.78 (0.7)	6.33 (0.92)	0.21**	6.7 (1.45)	5.35 (1.25)	6.37 (0.91)	0.15
Monocytes	0.56 (0.08)	n/ap	0.64 (0.12)	n/ap	0.44 (0.08)	0.31 (0.06)	0.57 (0.08)	0.15*	0.68 (0.14)	0.58 (0.13)	0.69 (0.14)	0.16
**Monocyte Expression CD64**	**MFI (SEM)**	**MFI (SEM)**	**MFI (SEM)**	**MFI (SEM)**	**MFI (SEM)**	**MFI (SEM)**	**MFI (SEM)**	**R squared**	**MFI (SEM)**	**MFI (SEM)**	**MFI (SEM)**	**R squared**
All Monocytes	29864.6 (6996.3)	n/ap	20991.8 (3449.8)	n/ap	15672.4 (2336)	18079.9 (3260.5)	17854.2 (2483.3)	0.22**	37554.1 (14014.2)	17230.8 (2379.1)	11511 (3063.1)	0.47****

Classical	31076.5 (7300.6)	n/ap	22083 (3509.7)	n/ap	16943.2 (2504.7)	19200.4 (3339.4)	18766.4 (2576.2)	0.20**	38999.7 (14638.8)	18002 (2518)	12182.9 (3184.8)	0.46****
Intermediate	29456.7 (8477.3)	n/ap	15004.7 (3073.9)	n/ap	9918.9 (1137)	13588.5 (3127.1)	16993.7 (3651.2)	0.28***	38419.8 (16546.3)	12222.8 (1432)	9435.5 (2794.6)	0.41****
Non-Classical	11566.6 (2950.4)	n/ap	7874.3 (1646.3)	n/ap	4605.3 (561.1)	6544.6 (2258.6)	7459.8 (1771.4)	0.17**	15729.7 (5805.1)	6897.5 (1152.1)	4130.9 (1154.1)	0.36****

Absolute counts are presented as mean and standard error of the mean (SEM) as x 10^9 cells/L of blood. Asterisks beside R squared indicate significance of the Welch's One-Way ANOVA. * P<0.0332, **P<0.0021, ***P<0.0002, ****P<0.0001. Monocyte expression presented as mean fluorescence intensity and SEM.

† Participants needing salvage therapy during the follow up phase were removed from analysis at 6 and 12 months.

‡ Complete blood count data collected at 4 weeks, immunophenotype data collected at 4.5 weeks.

Supplemental Figure 2: CBC Values and Normal Ranges. Each colour represents a different patient on the trial. Data points after starting salvage treatment were excluded from this figure. Normal ranges for our participant population are shown with dotted lines in each figure. Leukocyte Count, Platelet Count and Hemoglobin levels were monitored for patient safety. Study

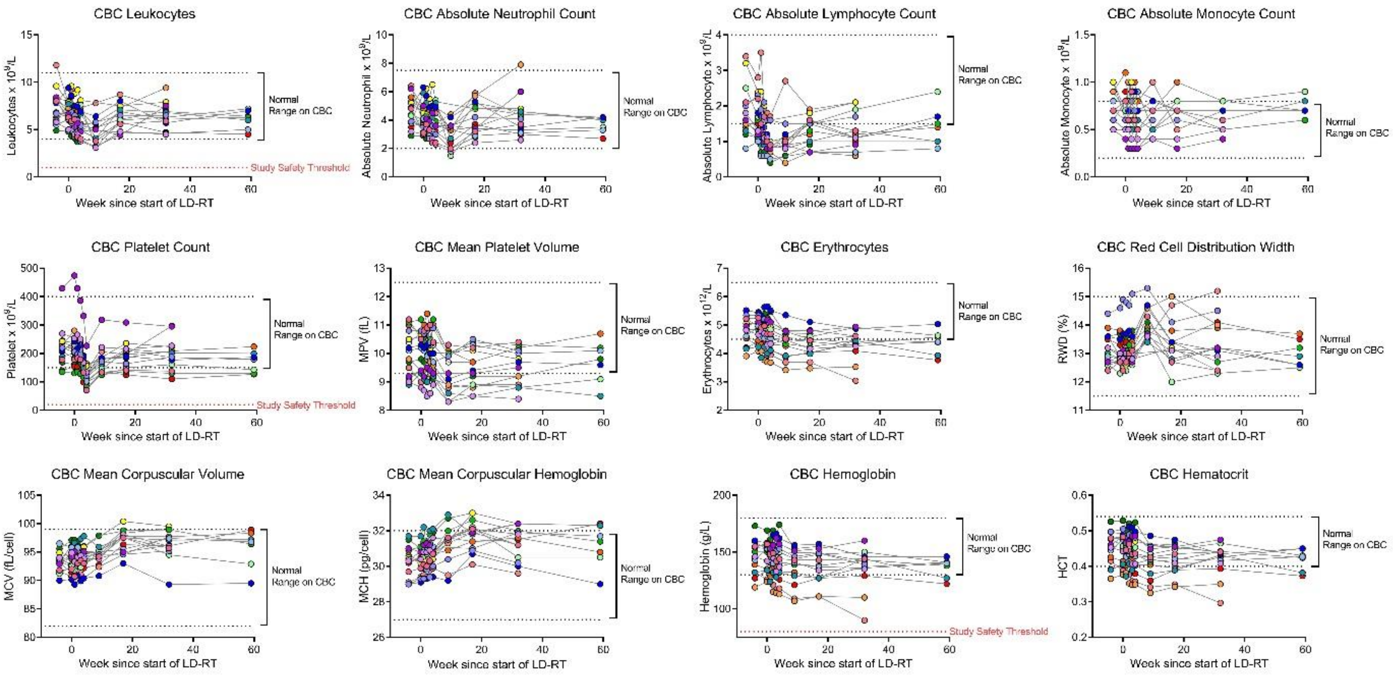

threshold is outlined in red for those parameters.
